# Fine-tuned continuous renal replacement therapy with calcium-free dialysate to manage severe hypercalcemia refractory to medical and intermittent hemodialysis

**DOI:** 10.1186/s40001-022-00715-x

**Published:** 2022-06-08

**Authors:** Marc Scheen, Grzegorz Nowak, Bienvenido Sanchez, Daniel Teta

**Affiliations:** 1 Service of Nephrology, Hôpital du Valais, Avenue Grand-Champsec 80, 1951 Sion, Switzerland; 2 Service of Intensive Care Medicine, Hôpital du Valais, Avenue Grand-Champsec 80, 1951 Sion, Switzerland

## Abstract

Malignancy-related hypercalcemia is a leading cause of hypercalcemia among hospitalized patients that carries poor prognosis. Parathyroid carcinoma is a rare form of primary hyperparathyroidism that may be associated with PTH dependent hypercalcemia. Severe hypercalcemia is life-threatening and may require management in an intensive care unit by means of medical therapy consisting of volume expansion, loop diuretics, cinacalcet, calcitonin and bisphosphonates. Renal replacement therapy such as intermittent hemodialysis has been successfully used among patients with severe hypercalcemia who become refractory to medical treatment. However, little data are available for cases of severe refractory hypercalcemia that fail to respond to both optimal medical therapy and hemodialysis. Our present case illustrates the successful use of continuous veno-venous hemodiafiltration (CVVHDF) with calcium-free dialysate calcium and markedly increased dialysate flow rate, to restore normal calcemia in a patient with metastatic parathyroid carcinoma with severe refractory hypercalcemia.

## Introduction

Malignancy-related hypercalcemia is one of the leading causes of hypercalcemia among hospitalized patients. Its presence is associated with poor prognosis [1]. Parathyroid carcinoma is rare and accounts for less than 1% of primary hyperparathyroidism. Its incidence peaks between the ages of 45 and 59 years, with men and women being represented equally in terms of incidence [2]. Severe hypercalcemia is a life-threatening electrolyte disorder that often requires management in an intensive care unit (ICU) [1]. It is mostly managed by medical therapy consisting of intravenous volume expansion, diuretics, calcitonin, bisphosphonates, denosumab, cinacalcet and corticosteroids [1, 3]. Only a few cases become refractory to medical therapy and eventually require renal replacement therapy (RRT) [1, 3–7]. Both continuous and intermittent forms of RRT have been used to manage severe hypercalcemia [3–7]. We report an exceptional case where medical therapy and intermittent hemodialysis failed to correct hypercalcemia due to parathyroid carcinoma. We successfully used continuous veno-venous hemodiafiltration (CVVHDF) with calcium-free dialysate calcium and markedly increased dialysate flow rate, to restore normal calcemia.

## Case presentation

**Fig. 1:**
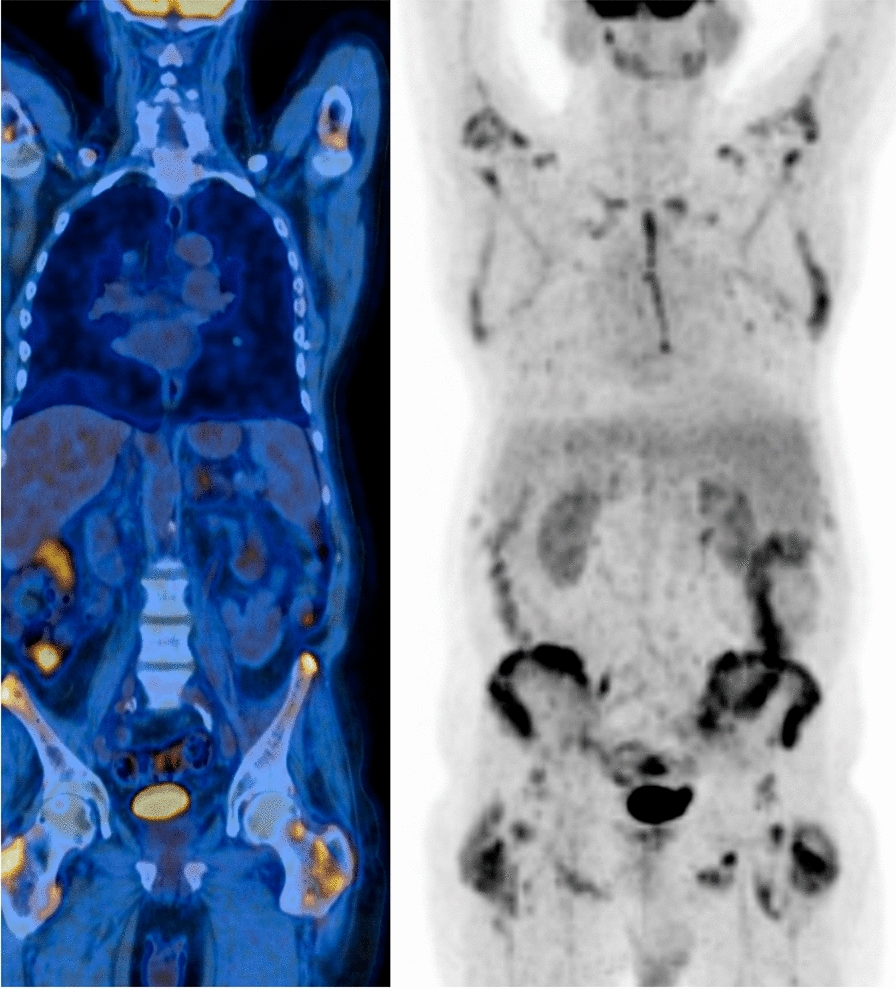
PET–CT and bone scan 1 month prior to admission showing tumor burden with pelvic bone lesions and metastatic lung disease

A 65-year-old Caucasian male patient, with a 3-year history of metastatic parathyroid carcinoma (lung, mediastinal, pelvic and sternal osteolytic bone lesions) and chronic kidney disease (CKD) stage G3bA1, was transferred to the ICU in June 2019, for severe, symptomatic hypercalcemia. His oncological history includes a right inferior parathyroidectomy with right hemithyroidectomy and cervical lymphadenectomy performed in 2015 followed by right thoracotomy and subsequent pulmonary wedge resection of the median lobe with inferior lobe metastasectomy in 2017 upon the discovery of metastatic disease during follow-up. Right posterolateral thoracotomy with pulmonary wedge resection of the right inferior lobe was performed in April 2019, 1 month before his ICU admission (Fig. 1). The patient had been admitted 4 days prior to the internal medicine ward hemodynamically stable, with normal arterial saturations, no fever and polyuria estimated at 8250 ml /24 h. Biological studies revealed an initial ionized and corrected calcium concentration of 2.46 mmol/l and 4.72 mmol/l, respectively, and an intact parathormone (PTH) plasma level of 499 pmol/L. He initially received intravenous volume expansion consisting of normal saline (3000 ml of NaCl 0.9% on day 1 and 7000 ml/24 h on days 2 to 4), oral prednisone 40 mg q24h, calcitonin (2100 UI cumulated over 72 h), a single dose of zoledronate 4 mg IV, and a progressively increased doses of cinacalcet (30 mg q12h during first 4 days) before his transfer in the ICU.

On admission to the ICU, the patient showed signs of volume depletion with hypotension, tachycardia and a urine output of 12 450 ml/24 h with a negative fluid balance. Arterial saturations were normal and no oxygen therapy or mechanical ventilation were required. A 12-lead ECG revealed atrial flutter with rapid ventricular response rate. We found mild metabolic acidosis, an ionized calcium of 1.99 mmol/l and plasma creatinine of 200 μmol/L, unchanged from baseline. A table comparing biological parameters on admission between the ICU and the medical ward is present in Table 1. Medical therapy was optimized with further normal saline increased to 12 500 ml/24 h in order to restore intravascular volume. No vasopressors were necessary due to the adequate response to volume expansion. Cinacalcet doses were increased to 120 mg q8h. Once the fluid balance became positive, loop diuretics were introduced in order to enhance calciuria. An intravenous bolus of furosemide 80 mg followed by an IV continuous infusion were titrated to obtain a neutral fluid balance. The final dose of subcutaneous calcitonin was administered 24 h after admission to the ICU as well as a single dose of 60 mg of denosumab. Despite these measures, ionized calcium continued to increase. Spot-quantified calcinuria corrected for creatininuria, while on diuretics, was of 4.05 mol/mol on day 10 of ICU admission.

**Table 1:** Biological studies on day 1 at the internal medicine ward and the ICU

	Day 1: internal medicine ward	Day 1: ICU
Potassium (mmol/l)	3.7	3.0
Ionized calcium (mmol/l)	2.46	1.99
Bicarbonates (mmol/l)	20.7	16.8
*p*H	7.35	7.32
*p*CO_2_ (kPa)	5.2	4.4
Hemoglobin (g/l)	93	90

**Fig. 2:**
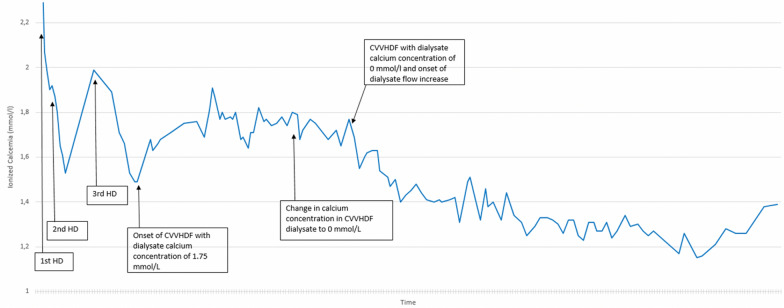
The “HD” arrows showing the three intermittent hemodialysis (HD) sessions initiated in the ICU, with the post-dialysis rebound within hours of termination. CVVHDF with standard pre, post and dialysate flow rates was then introduced with a dialysate, pre- and post-dilution calcium concentration of 1.75 mmol/l. CVVHDF was fine-tuned by optimizing calcium concentrations, lowering them to 0 mmol/l in both the dialysate and predilution solution. The final change involved an increase in dialysate flow rate up to 1500 ml/h. The synergic effect of lowering dialysate and predilution calcium concentrations with the increased dialysate flow rates on normalizing calcemia is made evident (final arrow)

Intermittent hemodialysis (IHD) sessions of 4 h, with low calcium dialysate, were initiated 48 h after ICU admission due to the difficulties encountered in controlling calcemia with medical therapy. Initiation of IHD was not done on the basis of acute kidney injury (AKI)-related complications (uremia, acidosis, hyperkalemia, etc.). It was carried out using a standard FX60^®^ polysulfone filter with a systemic unfractionated heparin-based anticoagulation. The dialysate calcium concentration was set to 1.25 mmol/l with blood flow rates of 300–400 ml/min and dialysate flow rates of 500 ml/min. A total of 3 intermittent hemodialysis were performed 24 h apart (Fig. 2) with the 3rd session lasting 5 h. A gradual decrease of calcium levels was observed after each hemodialysis session, however with rapid post-hemodialysis rebound hypercalcemia that reached pre-admission levels.

Continuous renal replacement therapy (CRRT) with unfractionated heparin systemic anticoagulation was then initiated on day 5 after ICU admission (Fig. 2). Parameters of continuous veno-venous hemodiafiltration (CVVHDF) were set according to local ICU protocol (Prismaflex^®^, ST150 Filter), with pre and post-dilutions, as well as dialysate calcium concentration of 1.75 mmol/l. Arterial blood samples for ionized calcium were frequently measured. The blood flow rate was initially set to 250 ml/min, with pre-, post-dilution and dialysate flow rates at 750 ml/h. After 24 h, the predilution and dialysate calcium concentrations were lowered to 0 mmol/l, and dialysate flow rate was progressively increased to 1500 ml/h. Only after this last measure, ionized calcium concentrations significantly decreased in a sustained manner. CRRT was terminated after 48 h of stable physiological calcemia. Serum calcium changes according to our complete management strategy are shown in the figure. No sodium citrate regional anticoagulation was used.

The patient was transferred back to the internal medicine ward, where maximal medical therapy was pursued and normal to borderline/high calcemia were maintained during several weeks. Pulmonary metastasectomy was performed during this interval with two additional doses of denosumab being administered. Unfortunately, hypercalcemia relapsed and re-admission in the ICU was necessary. During the second ICU stay, he underwent CVVHDF again using the same parameters and methodology, which normalized calcemia for the second time.

## Discussion

The majority of total body calcium (99%) is found in bone under the form of hydroxyapatite, with 1% occupying the extracellular and intracellular fluid [1]. Approximately 50% of extracellular calcium is protein bound, predominantly to albumin, with the remainder being diffusible as free ionized calcium or bound to anions such as phosphate or citrate [1]. Total body calcium as well as plasma calcium concentrations are maintained within physiological range by an interplay between enteric absorption, glomerular filtration with tubular reabsorption and the equilibrium between bone formation and bone resorption [1]. This interplay comes under hormonal control with the important regulatory role of PTH, 25-OH-D3, 1.25-OH-D3 and the FGF-23/Klotho system [1]. In our case, heavy PTH mediated bone resorption was probably responsible for an elevated endogenous release of calcium from bone. Indeed, we were unable to surgically control the high levels of PTH originating from metastatic ectopic foci of the parathyroid carcinoma in the lung parenchyma, mediastinal lymph nodes and bone. Low glomerular filtration rate associated with CKD may also have contributed to limiting renal excretion of calcium.

This article shows an example of a challenging case of metastatic parathyroid carcinoma with severe symptomatic hypercalcemia resistant to maximal medical and surgical treatments. Intermittent hemodialysis was ineffective due to post-dialysis rapid rebound hypercalcemia, consecutive to continuous endogenous malignant production. The current state of literature does not provide guidelines to manage such cases where standard medical and RRT fail. We employed CVVHDF with defined fine-tuned parameters. In particular, we demonstrated that normocalcemia was only achieved with dialysate calcium concentration set to 0 mmol/l, and a markedly high dialysate flow rate at 1500 ml/h. Although no complications occurred, the efficiency and safety of our strategy should be interpreted with caution and may not extrapolated to all cases of severe refractory hypercalcemia. In our patient, the net balance between production and clearance of calcium was difficult to predict. Factors such as polyuria, parallel medical and surgical treatments, unpredictable production of ectopic PTH as well as metastatic bone resorption all played a role in the calcium balance. Our patient was subject to extreme levels of malignant endogenous production of calcium. In this case, severe refractory hypercalcemia may perhaps have been prevented, if the primary sites of production had been removed earlier and the source of production controlled. However, we demonstrated that fine-tuned CVVHDF, with zero calcium in dialysate and a very high dialysate flow rate, may be successfully used as a bridge, while waiting for maximal medical and surgical treatments to become effective.

Sodium citrate regional anticoagulation was not used in our patient as it was considered unsafe. Our team of nephrologists and intensivists believed that high doses of citrate required to chelate extremely high calcium levels could have led to citrate toxicity. Furthermore, the post-filter calcium substitution became a subject of debate. With the use of a jugular central venous catheter as our vascular access, we estimated that using no post-filter calcium substitution, with a 0 mmol/l post-filter outflow, directly into the cardiac chambers may trigger arrhythmia. We are aware that we lack evidence to back our actions. However, there is no data supporting the safety of sodium citrate in extreme hypercalcemia. Furthermore, the safety of post-filter calcium equilibration inside the cardiac chambers in patients where the post-filter calcium is set to zero, has not been addressed.

The possibility of using a calcium-free dialysate during IHD was discussed by our nephrology team, but was also deemed to be too risky. The high arythmogenic potential of inducing an abrupt fall of calcium using high blood and dialysate flow rates in IHD despite it not having been reported by a couple of Korean case series [7]. Another potential solution may be to employ IHD with a zero calcium dialysate using low blood flow rate, titrated to clinical and biological response. This would theoretically be safe if frequent blood gas samples are drawn but was not considered, given the intense rebound hypercalcemia we faced upon interrupting IHD.

## Data Availability

Not applicable. The results presented in this paper have not been published previously in whole or part, except in abstract format.
